# Personalisierte Psychotherapie der posttraumatischen Belastungsstörung: Übersicht zur Auswahl von Behandlungsmethoden und -techniken mittels statistischer Verfahren

**DOI:** 10.1007/s00115-023-01549-6

**Published:** 2023-09-27

**Authors:** Carolin Steuwe, Jakob Blaß, Sabine C. Herpertz, Martin Drießen

**Affiliations:** 1 Universitätsklinik für Psychiatrie und Psychotherapie, Ev. Klinikum Bethel, Universitätsklinikum OWL der Universität Bielefeld, Remterweg 69–71, 33617 Bielefeld, Deutschland; 2 Klinik für Allgemeine Psychiatrie, Universitätsklinikum Heidelberg, Heidelberg, Deutschland

**Keywords:** Therapieauswahl, Behandlungsoptimierung, Machine Learning, Behandlungserfolg, Präzisionsmedizin, Treatment selection, Treatment optimization, Machine learning, Treatment outcome, Precision medicine

## Abstract

**Hintergrund:**

Eine relevante Heterogenität von Behandlungseffekten bei der posttraumatischen Belastungsstörung (PTBS) wird vor dem Hintergrund der Debatte um die Notwendigkeit einer phasenbasierten Behandlung sowie in Anbetracht der neuen Diagnose der komplexen PTBS diskutiert und ist inzwischen nachgewiesen. Dennoch gibt es bisher wenig Personalisierung in der Therapie der PTBS. Ziel der vorliegenden Arbeit ist es, den aktuellen Forschungsstand zur personalisierten Auswahl bestimmter Psychotherapiemethoden zur Behandlung der PTBS anhand von Patient:innenmerkmalen durch statistische Verfahren darzustellen.

**Methodik:**

Es wurde eine systematische Literaturrecherche in den Datenbanken PubMed (inkl. Medline), Embase, Web of Science Core Collection, Google Scholar, PsycINFO und PSYNDEX zu klinischen Studien und Übersichtsarbeiten zu personalisierter Therapie der PTBS durchgeführt.

**Ergebnisse:**

13 relevante Publikationen wurden identifiziert, hiervon waren 5 Artikel Prädiktoranalysen in Stichproben ohne Kontrollbedingung. 7 Artikel zeigten Analysen von randomisiert-kontrollierten Studien (RCT) mit einem Post-hoc-Vergleich der Behandlungseffekte optimal und nichtoptimal zugeordneter Patient:innen. Zudem liegt ein Review-Artikel zur Behandlungsauswahl bei PTBS und komorbider BPS vor.

**Diskussion:**

Die vorliegenden Publikationen deuten auf Bedeutung und Nutzen personalisierter Behandlung bei der PTBS hin. Die für die Personalisierung ermittelten relevanten Prädiktorvariablen sollten als Anregung genutzt werden, diese in prospektiven Studien genauer zu untersuchen.

## Abstract (en)

**Background:**

A relevant heterogeneity of treatment effects in posttraumatic stress disorder (PTSD) is discussed with respect to the debate about the necessity of phase-based treatment and in light of the new diagnosis of complex PTSD and has recently been proven; however, there has been little personalization in the treatment of PTSD. This article presents the current state of research on the personalized selection of specific psychotherapeutic methods for the treatment of PTSD based on patient characteristics using statistical methods.

**Methods:**

A systematic literature search was conducted in the PubMed (including Medline), Embase, Web of Science Core Collection, Google Scholar, PsycINFO and PSYNDEX databases to identify clinical trials and reviews examining personalized treatment for PTSD.

**Results:**

A total of 13 relevant publications were identified, of which 5 articles were predictor analyses in samples without control conditions and 7 articles showed analyses of randomized controlled trials (RCT) with a post hoc comparison of treatment effects in optimally and nonoptimally assigned patients. In addition, one article was a systematic review on the treatment of patients with comorbid borderline personality order and PTSD.

**Discussion:**

The available manuscripts indicate the importance and benefits of personalized treatment in PTSD. The relevant predictor variables identified for personalization should be used as a suggestion to investigate them in future prospective studies.

## Einleitung

Die Personalisierung von Psychotherapie dient dazu, Behandlungsentscheidungen auf Grundlage individueller Charakteristika der Behandelten zu treffen. In der Literatur werden die Begriffe Personalisierung und Individualisierung zum Teil synonym verwendet (z. B. [6]), die Auswahl der Begrifflichkeit wird derzeit kontrovers diskutiert [24], ohne dass bisher eine allgemein akzeptierte Definition oder Operationalisierung vorliegt. Beide Begriffe stehen dem der somatischen Präzisionsmedizin nahe. Im Folgenden wird der Einheitlichkeit wegen der Begriff der Personalisierung verwendet. Dieser beschreibt eine Behandlung unter Berücksichtigung des individuellen Geno- und Phänotyps und von Umweltbedingungen und Lebensstil [25]. Eine Personalisierung von Behandlung ist dann sinnvoll, wenn Patient:innen einer Diagnosegruppe unterschiedlich gut von einer psychotherapeutischen Behandlung profitieren. Ursachen für die interindividuelle Varianz der Wirksamkeit sind u. a. überdauernde, stabile Eigenschaften, wie z. B. demografische Variablen [33].

Bei der PTBS wurde eine substanzielle Varianzheterogenität der Behandlungseffekte in diagnosespezifischen randomisiert-kontrollierten Psychotherapiestudien (RCT), insbesondere im Vergleich von traumafokussierten Behandlungen und Wartelistenkontrollbedingungen, kürzlich nachgewiesen [2] und verdeutlicht den möglichen Nutzen personalisierter Ansätze. Heterogene Behandlungseffekte werden insbesondere bei der PTBS kontrovers diskutiert. Beispielsweise wird der Einsatz traumakonfrontativer Verfahren in Subgruppen mit bestimmten Merkmalen (z. B. mit selbstverletzendem Verhalten, Dissoziationen) kritisch hinterfragt oder gar für kontraindiziert/schädigend gehalten [13], während andererseits in einer (zu langen) Stabilisierungsphase das Risiko einer unnötig verzögerten oder ausbleibenden Remission gesehen wird [21]. Auch vor dem Hintergrund der in der ICD-11 eingeführten Diagnose der komplexen PTBS [31] wird die Wirksamkeit traumakonfrontativer Techniken und die Notwendigkeit zusätzlicher Behandlungselemente ganz aktuell diskutiert.

Trotz einer Vielzahl möglicher Unterscheidungsmerkmale (z. B. Art des traumatischen Ereignisses) und unterschiedlicher Behandlungstechniken (z. B. Exposition in sensu/in vivo, Stabilisierungstechniken, kognitive Umstrukturierung) werden personalisierte Behandlungsentscheidungen bisher zumeist auf Grundlage klinischer Intuition/Erfahrung getroffen.

Cohen et al. [5] differenzieren Personalisierungsentscheidungen im Kontext psychotherapeutischer Behandlungen im 3DP-Modell entlang von drei Dimensionen (Zeit, Struktur und Intervention; siehe Tab. 1). Die Zeitebene beschreibt den Behandlungsabschnitt, in dem eine personalisierte Behandlungsentscheidung getroffen wird. Die Interventionsebene beschreibt den Grad der Spezifizierung von Behandlungsentscheidungen (von der Makro- zur Mikroebene). Die Strukturebene beschreibt die Methode, mit der Behandlungsentscheidungen getroffen werden, bzw. deren Grad an Formalisierung. Seit Langem bekannt ist, dass statistische Vorhersagealgorithmen der klinischen Einschätzung überlegen sind [1, 28].

**Table 1:** 

Zeitebene	Interventionsebene	Strukturebene
1: Vor Behandlungsbeginn	1: Auswahl von Behandlungsumfang und -intensität	1: Idiosynkratisch/klinische Einschätzung
2: Während der Behandlung	2: Auswahl des Behandlungsverfahrens	2: Leitlinie
3: Nach der Behandlung	3: Auswahl spez. Behandlungsmethoden und -techniken	3: Entscheidungshilfe
	4: Anpassung des Vermittlungs- oder Interaktionsstils	4: Entscheidungsregel
		5: Statistisches Modell

Dieser Artikel beschäftigt sich mit der Auswahl von Behandlungsmethoden, -techniken oder -intensität (Interventionsebene) vor Behandlungsbeginn (Zeitebene) durch statistische Methoden (Strukturebene). Am Modell ist zu erkennen, dass es sich hierbei um einige unter mehreren wichtigen Behandlungsentscheidungen handelt.

Zur präzisen statistischen Vorhersage der individuellen Wirksamkeit einer spezifischen Intervention können traditionelle, hypothesengeleitete und neuere datengetriebene Verfahren (Machine Learning [ML]) unterschieden werden.

Aus den Nachteilen traditioneller Prädiktoranalysen (hohe notwendige Fallzahlen, geringe Anzahl eingeschlossener potenziell relevanter Variablen, „overfitting“ und seltene Replikationen) entstand der Wunsch nach multivariaten Ansätzen, die soziodemografische, klinische, biologische und psychometrische Daten in einem Modell gemeinsam erfassen [3]. ML-Ansätze sind nicht an Hypothesentests gebunden, sondern haben zum Ziel, Prädiktionsmodelle zu erstellen, die zuverlässige Vorhersagen in neuen Datensätzen ermöglichen. Bei diesem datengetriebenen Vorgehen können viele Prädiktorvariablen kombiniert werden, da durch verschiedene Techniken der Gefahr des „overfitting“ entgegengewirkt wird. Mit steigender Anzahl an Prädiktorvariablen erhöht sich jedoch auch hier das Risiko von „overfitting“ [3]. Gerade komplexe Modelle erfordern große Datenmengen, um traditionellen statistischen Verfahren tatsächlich überlegen zu sein [11]. Eine externe Validierung der Modelle in unabhängigen Daten ist unabdingbar [3].

Ziel der vorliegenden Arbeit ist es, den aktuellen Forschungsstand zur statistischen, personalisierten Auswahl bestimmter Behandlungsmethoden und -techniken anhand von Patient:innenmerkmalen und den durch Personalisierung potenziell erreichbaren Vorteilen in der Behandlung der PTBS darzustellen. Dabei sollen traditionelle Analysen und ML-Algorithmen berücksichtigt werden.

## Methodik

Im Rahmen einer systematischen Literaturrecherche in den Datenbanken PubMed (inkl. Medline), Embase, Web of Science Core Collection, Google Scholar, PsycINFO und PSYNDEX am 12.10.2022 und 30.06.2023 wurden Studien und Übersichtsartikel zur personalisierten Behandlung der PTBS identifiziert, bei denen durch statistische Methoden (erwartete) Effekte einer personalisierten Auswahl spezifischer Methoden und Techniken (Patient:in-Behandlung-Interaktion) untersucht wurden^1^. Dies erfordert, dass mindestens zwei unterschiedliche Behandlungsbedingungen verglichen werden. Die Interpretierbarkeit von komplexen ML-basierten Modellen ist in den diesbezüglich vorliegenden Daten limitiert, weshalb folgende Studien eingeschlossen wurden:

- Traditionelle Analysen mit Schätzung der Effekte einer personalisierten Zuweisung mindestens zweier Behandlungsverfahren
- ML-basierte Analysen zur Schätzung der Effekte einer personalisierten Zuweisung mindestens zweier Behandlungsverfahren
- ML-basierte Analysen mit Vorhersage des Erfolgs nur einer spez. Psychotherapiemethode

Die Suche wurde limitiert auf Manuskripte, die auf Deutsch oder Englisch veröffentlicht wurden und folgende Suchbegriffe beinhalteten: (personaliz* OR personalis* OR optimiz* OR optimis* OR individualiz* OR individualis*) AND (PTSD OR posttraumatic stress disorder) AND (treatment OR therapy).

**Figure 1:**
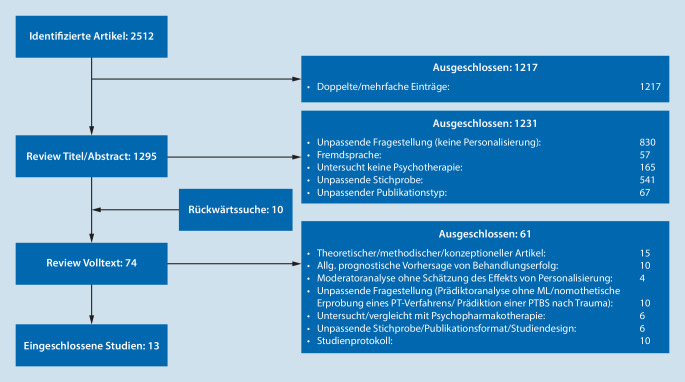

Im ersten Schritt wurden Titel und Abstracts aller Funde gesichtet. Thematisch und methodisch unpassende Funde wurden ausgeschlossen. Hierzu gehörten Studien, die keine Psychotherapie untersuchten, Einzelfallstudien, Studien mit dem Ziel eines nomothetischen Wirksamkeitsnachweises (z. B. Onlinetools mit persönlicher vs. automatisierter Rückmeldung), allgemeine Übersichtsartikel zur PTBS oder anderen psychischen Erkrankungen oder Studien, in denen eine andere Stichprobe als erwachsene PTBS-Patient:innen behandelt wurde. Von den eingeschlossenen Studien wurden Volltexte gesichtet. Ausgeschlossen wurden rein methodische/hypothesenbildende Schriften und traditionelle Prädiktoranalysen ohne ML oder Schätzung der Effekte personalisierter Behandlung sowie Studien, die Variablen zur Vorhersage nutzten, die in der Routineversorgung nicht praktikabel sind (z. B. Biomarker, funktionelles Neuroimaging). Darüber hinaus wurden Studien ausgeschlossen, in denen rein prognostische Aussagen über Therapieerfolg bei PTBS, d. h. über unterschiedliche psychotherapeutische Behandlungsmethoden und -techniken hinweg, getroffen wurden. Zusätzlich wurde in allen Volltexten eine Rückwärtssuche zu Arbeiten durchgeführt, welche den o. g. Kriterien entsprachen (Abb. 1).

## Ergebnisse

Es wurden 13 relevante Publikationen identifiziert: 7 Artikel zeigen RCT mit Post-hoc-Vergleich optimal und nichtoptimal behandelter Patient:innen (davon 2 mit traditionellen statistischen Verfahren), 5 Artikel zeigen ML-basierte Prädiktoranalysen für spez. Behandlungsmethoden und -techniken. Zudem liegt ein Review-Artikel zur Behandlungsauswahl bei PTBS und komorbider Borderline-Störung vor.

### Methodik der Prädiktor‑/Personalisierungsstudien

#### Identifikation von Prädiktorvariablen.

Die Prädiktorauswahl erfolgte in den Studien mit traditionellen statistischen Verfahren hypothesengeleitet. Bei den ML-basierten Studien wurden unterschiedliche ML-Ansätze zur Prädiktorauswahl genutzt. Diese Ansätze haben gemein, ohne vorherige Hypothesen bzgl. Prädiktorvariablen zu ermitteln, welche Variablen bedeutsam zur Vorhersage des Outcomes sind. Dabei geht es nicht darum, einen vorliegenden (Trainings‑)Datensatz am besten erklären zu wollen, sondern die Vorhersage in einem unabhängigen (Test‑)Datensatz zu optimieren. Hierfür wird eine Vielzahl von Substichproben erzeugt, in denen Prädiktoren ermittelt und durch wiederholte Prädiktion in weiteren Substichproben auf replizierbare Wichtigkeit für das Vorhersagemodell geprüft werden. Die zum ML gewählten Algorithmen werden hier nicht im Detail geschildert, sind aber in Tab. 2 und 3 dargestellt.

**Table 2:** 

	Behandlungserfolg bei traumafokussierten Behandlungsmethoden				
*Studie/Autoren*	Nixon et al., 2021 [23]	Herzog et al., 2021 [16]	Held et al., 2022 [14]	Smith & Held, 2022 [14]	Stuke et al., 2021 [27]
*Größe der Stichprobe*	*n* = 179	*n* = 612	*n* = 432	*n* = 362	*n* = 116
*Outcome*	PTBS-Symptomschwere: Ansprechen auf Therapie def. durch Reliable Change Index, Erfüllen der Diagnose u. selbstberichtete Symptome	PTBS-Symptomschwere und Funktionsniveau	PTBS-Symptomschwere: Ansprechen auf Therapie ermittelt durch Mischverteilungsmodelle („fast“ und „minimal response“)	PTBS-Symptomschwere	PTBS-Symptomschwere
*Population*	Frauen mit interpersonellen traumatischen Erfahrungen	Deutsche Regelversorgung	Veteran:innen (Überschneidung mit Smith & Held)	Veteran:innen (Überschneidung Held et al.)	Deutsche Regelversorgung
*Intervention*	Cognitive Processing Therapy	Traumafokussierte kognitive Verhaltenstherapie (stationär)	Cognitive-processing-therapy-basierte intensive Behandlung	Cognitive-processing-therapy-basierte intensive Behandlung	Cognitive Processing Therapy (teilstationär)
*Ergebnisse der Intervention*	Gutes Ansprechen bei 81 % der Pat.	Sig. Reduktion der PTBS-Symptomschwere (d = 0,6)	Sig. Reduktion der PTBS- und Depressionssymptomschwere	Sig. Reduktion der PTBS- und Depressionssymptomschwere	Sig. Reduktion der PTBS-Symptomschwere (d = 0,8–0,9)
*Ergebnisse*					
Algorithmus des Vorhersagemodells	Random Forest	Elastic Net	Elastic Net Gradient-Boosted Models Random Forest Ridge Classification Logistische Regression Logistische Regression mit Max-Min-parent-child-Variablenauswahl	Mixed Bayesian Additive Regression Trees Mixed Effects Random Forest Linear-mixed-effects-Regressionsmodelle	OLS Linear Regression Non-Linear Regression mit ADAboost Regressor
Prädiktoren	Keine Vorhersage durch Baseline-Variablen möglich, Verbesserung der Vorhersage durch Erfassung der PTBS-Symptomschwere im Behandlungsverlauf Allerdings: homogene Stichprobe und nur wenige Nonresponder:innen	Outcome PTBS-Symptomschwere: Höheres Lebensalter, Rentenwunsch/berentet sein, Gesamtzahl Diagnosen, Psychotizismus u. Depressivität → geringere Wirksamkeit Höhere Prä-PTBS-Symptomschwere (insbes. Vermeidung) → bessere Wirksamkeit Outcome Funktionsniveau: Psychotizismus, Asthma, Ängstlichkeit, Stoffwechselerkrankung → geringere Wirksamkeit; Gesamtanzahl Diagnosen → höhere Wirksamkeit	Fast response: Prä-PTBS-Symptomschwere und sex. Trauma während Militärzeit Minimal response: Prä-PTBS-Symptomschwere; demografische und Militärservice-bezogene Variablen mit geringer Wichtigkeit (allerdings hohe Homogenität der Stichprobe)	Baseline-Prädiktoren erklären 20 % der Varianz des Outcomes, davon 14 % durch Prä-PTBS-Symptomschwere und 6 % durch Demografie Verbesserung der Vorhersage durch Erfassung der PTBS-Symptomschwere im Behandlungsverlauf	Traumaassoziierte Variablen (Kognitionen, Ereigniszentralität), Depressivität → geringere Wirksamkeit Robustere Vorhersage durch Nutzung des Ansprechens auf Therapie nach 4 Wochen als Prädiktor
Klinische Implikationen	Entscheidung über Beendigung o. Wechsel der Behandlungsmethode sollte nicht voreilig getroffen werden Variablen, wegen derer zu Vorsicht zur Anwendung von traumafokussierter Beh. geraten wurde, hatten keine Vorhersagekraft	Prädiktoren erklären moderaten, aber klinisch relevanten Varianzanteil Anregung zur breiten Erfassung der genannten und weiterer Prädiktoren in der Regelversorgung (z. B. Therapieerwartungen, traumatische Erfahrungen in der Kindheit)	Vorhersage von Fast- und Minimal-Responder:innen mit moderater Genauigkeit möglich Traumaspez. Variablen mit höchster Vorhersagewichtigkeit Genauigkeit noch nicht ausreichend für Behandlungsentscheidungen	Bester Prädiktor ist Verbesserung im Therapieverlauf, nicht Baseline-Variablen	Prädiktion des Therapieerfolgs vor Beginn möglich, aber nur kleiner Anteil erklärter Varianz Verbesserung der Vorhersage durch Monitoring des Ansprechens auf Therapie nach 4 Wochen Klinische Empfehlungen noch nicht möglich

*PTBS* posttraumatische Belastungsstörung

**Table 3:** 

	Traumafokussierte vs. nichttraumafokussierte Therapie	Vergleich traumafokussierter Behandlungsmethoden			Sitzungsanzahl traumafokussierter Behandlung	Einsatz stabilisierender Elemente	
*Studie/Autoren*	Wiltsey Stirman et al., 2021 [30]	Deisenhofer et al., 2018 [8]	Cohen et al., in Vorbereitung [7]	Keefe et al., 2018 [18]	Resick et al., 2021 [26]	Cloitre et al., 2016 [4]	Hoeboer et al., 2021 [17]
*Größe der Stichprobe*	*n* = 267	*n* = 317	*n* = 159	*n* = 160	*n* = 127	*n* = 104	*n* = 149
*Outcome*	PTBS-Symptomschwere	Symptomschwere Depression (korreliert stark mit PTBS-Schwere)	PTBS-Symptomschwere	Abbruchrate	PTBS-Symptomschwere: Remission bzw. „good end-state criteria“ bzgl. PTBS	PTBS-Symptomschwere	PTBS-Symptomschwere
*Population*	Veteraninnen und Frauen im aktiven Militärdienst	Britische Regelversorgung	Frauen nach Vergewaltigung (selbe SP wie Keefe et al.)	Frauen nach Vergewaltigung (selbe SP wie Cohen et al.)	Frauen und Männer im aktiven Militärdienst	Frauen mit traumatischen Erfahrungen in der Kindheit	Männer und Frauen mit Missbrauchserfahrungen in der Kindheit
*Vergleichsbedingungen*	Prolonged Exposure (PE) vs. Present-Centered Therapy (PCT)	Traumafokussierte kognitive Verhaltenstherapie (tfKVT) vs. Eye Movement Desensitization and Reprocessing (EMDR)	Prolonged Exposure (PE) vs. Cognitive Processing Therapy (CPT)	Prolonged Exposure (PE) vs. Cognitive Processing Therapy (CPT)	Remission in ≤ 12 Sitzungen vs. Remission nach > 12 Sitzungen oder „non-response“ unter Cognitive Processing Therapy (CPT)	Prolonged Exposure (PE) + supportive Gespräche (SupC) vs. Skills Training in Affective and Interpersonal Regulation (STAIR) + SupC vs. STAIR + PE	Prolonged Exposure (PE) vs. Skills Training in Affective and Interpersonal Regulation (STAIR) + PE
*Ergebnisse der Primärstudie*	Stärkere Symptomreduktion unter PE (d = 0,27)	Keine Effektivitätsunterschiede zwischen EMDR und tfKVT	Keine Effektivitätsunterschiede zwischen PE und CPT	Keine Unterschiede in Abbruchraten zwischen PE und CPT	–	Kein Unterschied in Remissionsraten zwischen den Gruppen, Überlegenheit von STAIR + PE bei PTBS-Symptomschwere	Keine Effektivitätsunterschiede zwischen PE und STAIR + PE
*Ergebnisse*							
Algorithmus des ML-Vorhersagemodells	Elastic Net Bootstrapped Backward Elimination bei OLS Regression	Genetic Algorithm bei OLS Regression	Random Forest Elastic Net Bayesian Additive Regression Trees Bootstrapped Backward Elimination bei OLS Regression	Random Forest Bootstrapped Backward Elimination bei OLS Regression	–	–	Random Forest Bootstrapped Backward Elimination bei OLS Regression
Prädiktoren	Prognostisch (allg.): Hohe Prä-PTBS-Symptomschwere, sexuelle Gewalt im Militärdienst → schlechtere Prognose Gute körperliche Fitness, hohe subjektive Vertrauenswürdigkeit der Behandlung → bessere Prognose Pat. mit guter Prognose profitierten signifikant mehr von PE als von PCT, kein Unterschied bei Pat. mit schlechter Prognose	Präskriptiv: tfKVT: weibliches Geschlecht, Berufstätigkeit, höheres Lebensalter → höhere Wirksamkeit Geringeres Funktionsniveau → geringere Wirksamkeit EMDR: höhere Belastung zu Behandlungsbeginn, Einnahme von Antidepressiva → geringere Wirksamkeit	Prognostisch: PE: Dissoziationen → höhere Wirksamkeit Wütendes Temperament, Anzahl verschiedener Straftaten → geringere Wirksamkeit CPT: sex. Missbrauch in der Kindheit → geringere Wirksamkeit Wütendes Temperament, Störung d. Tag-Nacht-Rhythmus → höhere Wirksamkeit	Prognostisch (allg.): Anzahl an Schuljahren, geschätzter IQ-Wert → geringere Abbruchrate Präskriptiv: CPT: missbräuchliche Beziehung → geringere Abbruchrate PE: kaukasische Herkunft → geringere Abbruchrate	Prognostisch für mehr Sitzungen und Non-Response: Höhere PTBS- und Depressionssymptombelastung, afroamerikanische Abstammung, geringere Veränderungsmotivation, introvertiertes Temperament	Präskriptiv – Clusterung in Quartile nach Moderatorindex aus Symptomschwere und Emotionsregulation: STAIR + PE: kein Einfluss des Moderatorindex STAIR + SupC: kein Einfluss des Moderatorindex PE + SupC: höchster Moderatorindex (hohe Symptomschwere und Emotionsregulationsprobleme) → Zunahme der PTBS-Symptombelastung; niedrigster Moderatorindex → Reduktion der PTBS-Symptombelastung	Prognostisch: PE: schwere depressive Symptome, weniger soziale Unterstützung, mehr Achse-I-Komorbiditäten und schwerer sexueller Missbrauch in der Kindheit → geringere Wirksamkeit STAIR + PE: stärkere Emotionsregulationsstörungen, höhere PTBS-Symptombelastung, schlechtere körperliche Gesundheit → geringere Wirksamkeit
Anteil an Pat., die von Personalisierung profitiert hätten (nichtoptimal randomisiert)	31,5 % (*n* = 84)	44,9 % (*n* = 101)	Keine Angabe	52,5 % (*n* = 84)	Keine Angabe	67,6 % (*n* = 48)	50,0 % (*n* = 74) nach CAPS 37,0 % (*n* = 55) nach PCL
Erwartete Effekte von Personalisierung	Sign. Interaktion zw. prognostischem Index und Behandlungsart spricht für das Potenzial personalisierter Behandlung	Effektstärke von d = 0,4	Effektstärke von d = 0,4	Unterschied in den Abbruchraten (20 vs. 40 %)	Hinweise auf höhere PTBS-Remissionsrate (65 vs. 40 %) und bessere Behandlungsergebnisse (d = 0,63 für die Between-group-Effektstärke in Bezug auf das PTBS-Outcomemaß) bei variabler Sitzungszahl im Vergleich zu CPT-Studie mit fester Sitzungsanzahl	Differenzielle Effekte auf Behandlung in Abhängigkeit vom Moderatorindex erst zum Follow-up-Zeitpunkt Höhere Effektstärken bei optimaler Zuordnung	Höhere Effektstärken bei Pat., die zufällig ihrer optimalen Behandlung zugewiesen wurden

*PTBS* posttraumatische Belastungsstörung, *SP* Stichprobe

#### Methodik zur Identifikation von erwarteten Personalisierungseffekten.

7 Studien nehmen eine Schätzung der erwarteten Effekte von Personalisierung vor, davon sind 6 Sekundäranalysen. Eine Studie [26] mit traditioneller Statistik ermittelte prospektiv, ob Patient:innen von einer individuellen Anzahl von CPT-Sitzungen (zwischen 12 und 24) profitieren. Anschließend wurden relevante Prädiktoren für die Notwendigkeit einer Erhöhung der Therapiesitzungen ermittelt (z. B. für die Subgruppe mit Ansprechen nach wenigen Sitzungen). Da diese Studie nicht auf einer früheren Behandlungsstudie basiert, wurden Daten aus einer früheren Studie mit fester Sitzungszahl herangezogen, um den Effekt der Personalisierung zu schätzen. Hier ergaben sich methodische Schwierigkeiten durch Unterschiede der verglichenen Studien, sodass zur Verbesserung aktuell eine RCT mit fester vs. variabler Sitzungsanzahl durchgeführt wird [29]. Die zweite Studie mit traditioneller Analyse ermittelte aus hypothesengeleiteten Prädiktorvariablen einen Moderatorindex, der eine Subtypisierung der untersuchten Stichprobe anhand prädiktiver Merkmale vornimmt [4]. Nachfolgend wurde ein Interaktionseffekt zwischen Therapieeffekt und Moderatorindex bestimmt und Effekte der Personalisierung der Behandlung ermittelt, indem die Ergebnisse der Patient:innen mit zufällig optimaler Behandlungszuordnung mit denen der randomisierten Zuordnung verglichen wurden.

4 ML-basierte Studien [7, 8, 17, 18] nutzen den Personalized Advantage Index (PAI, [10]) zur Post-hoc Schätzung zu erwartender Effekte von Personalisierung. Die Methode baut auf der Identifikation von Prädiktorvariablen auf (s. oben) und schätzt das jeweilige Outcome (z. B. die Symptomschwere) bei Behandlungsende. Dabei können prognostische und präskriptive (zwischen Behandlungen differenzierend) Indikatoren von Behandlungserfolg bestimmt werden. Der PAI beschreibt die für jede Patient:in vorhergesagte Differenz im Outcome zwischen mindestens zwei Behandlungen [10]. Nachfolgend wird bestimmt, welcher Behandlungseffekt sich potenziell gezeigt hätte, wären die Patient:innen jeweils mit der für sie optimalen Methode behandelt worden. Zudem werden Unterschiede in der Symptomreduktion zwischen Patient:innen untersucht, die zu ihrer optimalen vs. nichtoptimalen Behandlung randomisiert wurden. Es wurde keine Studie gefunden, die ML-Vorhersagen prospektiv zur Personalisierung nutzt und diese mit einer Kontrollbedingung (z. B. Randomisierung) vergleicht. Eine Studie [30] ermittelt einen allgemeinen prognostischen Index (vs. PAI: Prognose für jeden Behandlungsarm), der aus den identifizierten Prädiktorvariablen (s. oben) gebildet wird. Aufbauend werden Interaktionseffekte zwischen Index und Therapiemethode ermittelt. Für Ergebnisse siehe Tab. 3.

### Prädiktoren von Behandlungserfolg, erwartete Effekte der Personalisierung und klinische Implikationen

Tab. 2 und 3 zeigen die wichtigsten ermittelten Prädiktoren der jeweiligen Behandlungsmethoden und Outcomes. Die relevanten Variablen in Prädiktor- und Personalisierungsstudien sind unterschiedlich und abhängig von den zu Beginn der Therapie erfassten Variablen. Am häufigsten zeigen sich Zusammenhänge zwischen Prä-PTBS-Symptomschwere und Outcome, die jedoch in ihrer Richtung unterschiedlich sind [14, 16, 17, 27, 30]. In mehreren Studien zeigte sich eine schlechtere Wirksamkeit der traumafokussierten Behandlung bei sexueller Traumatisierung [7, 14, 30] und Komorbiditäten, insbesondere einer depressiven Symptombelastung [8, 16, 17, 27]. Soziodemografika spielen, obwohl häufig erfasst, eine untergeordnete Rolle. Berufstätigkeit zeigt sich in zwei Studien als prognostisch günstiger Faktor [8, 16]. Ein schlechter körperlicher Gesundheitszustand [16, 17, 30] scheint prognostisch ungünstig zu sein.

Nur drei Studien [4, 7, 18] machen präskriptive Aussagen für die einzelnen Therapiemethoden. Dass in den Studien mit rein prognostischen Aussagen unterschiedliche Prädiktoren zwischen den Behandlungsmethoden gefunden wurden, weist jedoch auf das Vorliegen von präskriptiven Variablen hin.

Die Prädiktion des PTBS-Behandlungserfolgs auf Grundlage von Baseline-Variablen erscheint klinisch relevant, auch wenn nur ein Teil der Varianz (ca. 20 %) erklärt werden kann. Behandlungsentscheidungen sind aktuell auf Grundlage der vorliegenden Daten zur Behandlungszuordnung durch Baseline-Variablen noch nicht möglich. Alle PAI-Studien weisen auf einen möglichen Vorteil von Personalisierung für den Behandlungserfolg hin. Es zeigen sich mittlere bis hohe Effektstärken zwischen Patient:innen nach optimaler vs. nichtoptimaler Zuordnung.

### Personalisierung und Komorbidität

Komorbide psychische Störungen sind bei der PTBS häufig [19] und könnten relevante Prädiktorvariablen für den Therapieausgang bei PTBS sein. Einige der o. g. Studien legen nahe, dass Patient:innen mit PTBS und hoher depressiver Symptombelastung weniger gut von traumafokussierter kognitiver Verhaltenstherapie profitieren als PTBS-Patient:innen ohne depressive Symptombelastung (z. B. [16]). Zur Reihenfolge und spezifischen Behandlungsmethoden bei komorbider Depression gibt es noch keine Befunde. Zur Optimierung der Behandlung von komorbider PTBS und BPS wird in einer aktuellen Übersichtsarbeit [32] die Notwendigkeit der ML-gestützten Identifikation mehrerer Prädiktorvariablen betont, ohne dass spezifische Studien hierzu existieren.

## Resümee

Obwohl sich dieses Forschungsfeld noch in den Anfängen befindet, weisen die vorliegenden Publikationen auf Bedeutung und Nutzen personalisierter Behandlung bei PTBS hin. Die dabei ermittelten relevanten Prädiktorvariablen sollten in zukünftigen, prospektiven Studien genauer untersucht werden und können aktuell noch nicht der klinischen Entscheidungsfindung dienen. Patient:innenmerkmale, die mit einer Überlegenheit einer alleinigen Anwendung nichttraumafokussierter Methoden verbunden sind, konnten nicht identifiziert werden, hingegen gab es Merkmale, für die eine Kombination von Exposition und stabilisierenden Techniken im Vergleich zu Exposition alleine als wirksamer eingeschätzt wurde.

Der Behandlungsverlauf bei PTBS-Patient:innen ist also unterschiedlich. In der Vorhersage des Behandlungsverlaufs ergeben sich zwischen datengetriebenen und hypothesengeleiteten Prädiktoranalysen z. T. interessante Widersprüche [4, 17], bspw. bezüglich der Prognose für Patient:innen mit Emotionsregulationsproblemen in traumafokussierten Behandlungen mit und ohne Vermittlung zusätzlicher stabilisierender Skills. Während sich bei Cloitre et al. [4] zeigte, dass sich Emotionsregulationsstörungen (in Kombination mit der Symptomschwere) nicht negativ auf den Behandlungserfolg auswirken, wenn Exposition und Skills kombiniert werden, zeigten sich Emotionsregulationsstörungen bei Hoeboer et al. [17] auch in der Kombination von Exposition und Skills als negativer Prädiktor für den Behandlungserfolg.

In den PAI-Studien hätten durchschnittlich mehr als 40 % von einer personalisierten Zuweisung besser profitiert als von einer randomisierten Zuordnung. Beachtenswert ist, dass es sich hierbei um einen Zusatznutzen bei bereits hoch wirksamen Behandlungsmethoden handelt. Da bisher keine Studien vorliegen, in denen personalisierte und randomisierte Zuordnung direkt verglichen werden, können die Effekte einer Personalisierung nur geschätzt werden. Zudem sind die Stichproben, die bisherige PAI-Studien nutzen, zumeist zu klein, um verlässliche multivariate Modelle zu erstellen [20]. Zuletzt wurden daher insbesondere Prädiktoranalysen größerer Stichproben ohne den Anspruch einer Schätzung des personalisierten Behandlungserfolgs durchgeführt. Auskunft über präskriptive Prädiktorvariablen ist notwendig, um eine personalisierte Zuteilung zu unterschiedlichen Behandlungsmethoden vorzunehmen und nicht lediglich eine Vorhersage der Wirksamkeit einer spezifischen Methode vorzunehmen. Ein mögliches Vorgehen könnte das folgende sein:

1. Externe Validierung bzw. Aktualisierung der Vorhersagemodelle in ML-basierten prognostischen Studien zweier Behandlungsmethoden (ggf. in bereits vorhandenen Daten) zur Ermittlung stabiler Prädiktoren einer Behandlungsmethode
2. Prospektive, randomisierte Studie mit dem Hauptfaktor: zufällige oder klinische vs. personalisierte Zuordnung (z. B. mit PAI) einer Behandlungsmethode (Patient:innen und Therapeut:innen verblindet)
3. Errechnung des Haupteffekts der Art der Zuteilung zur Schätzung des Effekts von personalisierter Zuordnung

Delgadillo et al. [9] konnten den Erfolg eines solchen Vorgehens bei der Depression demonstrieren. Datengetriebene Analysemethoden können die empirische Basis auch auf der Ebene der Wirkmechanismen verbessern und zukünftig Entscheidungsalgorithmen hinsichtlich Auswahl und Reihenfolge von Behandlungsbausteinen im Sinne einer modularen Psychotherapie ermöglichen [12, 15]. Die vorliegenden Ergebnisse unterstreichen die Notwendigkeit, verschiedene Personalisierungsansätze, d. h. auch Therapieprozessforschung (z. B. Feedback-Forschung), gleichzeitig voranzutreiben und kombiniert zu untersuchen. Um die Ergebnisse verschiedener Personalisierungsansätze zu verbinden und ausreichend große Datenmengen zu erheben, sind Forschungskooperationen und die Nutzung großer naturalistischer Stichproben unerlässlich. Einige Autoren werben zudem für die Erfassung inflammatorischer, neurobiologischer und psychophysiologischer Faktoren als potenzielle Prädiktoren [22]. Aufgabe zukünftiger Forschung ist es, hier eine gute Balance zwischen hoher Varianzaufklärung und praktikabler Erhebung in der klinischen Routine zu finden.

## Fazit für die Praxis

- Fast alle Forschungsergebnisse stammen aus den letzten fünf Jahren.
- Personalisierung verspricht eine Verbesserung der Behandlung; bekannte wichtige Prädiktorvariablen umfassen insbesondere klinische Merkmale wie PTBS- und Depressionssymptomschwere, bedürfen aber noch weiterer Untersuchung.
- Es fehlen prospektive Analysen zur Personalisierung der PTBS-Behandlung.
- Zukünftig sollte die kombinierte Nutzung von Baseline- und Prozessinformationen zur Personalisierung erforscht werden.

## Footnotes

1 Eine vollständige Auflistung aller Studien und der ausgewählten Volltexte kann bei der Erstautorin angefragt werden.
